# DEVELOPING AND IMPLEMENTING A NATIONWIDE CURRICULUM FOR AGING STUDIES. WHAT ARE THE KEY CONSIDERATIONS FOR GHANA?

**DOI:** 10.1093/geroni/igad104.2600

**Published:** 2023-12-21

**Authors:** Grace Karikari, Samuel Asante

**Affiliations:** University of North Dakota, Grand Forks, North Dakota, United States; Northeastern State University, Broken Arrow, Oklahoma, United States

## Abstract

In 2019, Ghana reported the implementation of a Geriatric Medicine Fellowship Program within the Faculty of Family Medicine at the Ghana College of Physicians and Surgeons (GCPS). Though this is a laudable effort to address the specialized workforce shortage the country is facing, the GCPS program is meant to provide an in-country specialization path for physicians desiring to become geriatricians. There are currently no national standardized aging studies, training programs or curricula available to other students in disciplines such as nursing, psychology, public health, and social work. Students in the public, government-owned tertiary institutions are offered isolated courses related to aging and the needs of older adults. To effectively address the workforce shortage issue and improve the overall health and wellbeing of older adults in Ghana, it is imperative to train students in diverse fields and disciplines with the needed knowledge and skills to provide the various specialized services for the aging population. This presentation highlights a proposal to the government of Ghana to consider some key issues and recommendations related to developing and implementing a nation-wide aging studies programs in the public higher institutions. Five key evidence-based areas are extensively discussed: (1) Aging studies in Ghana should be culturally relevant. (2) Aging studies in Ghana should address the needs of older Ghanaians. (3) Aging studies in Ghana should be competency-based. (4) Aging studies in Ghana should be implemented and evaluated by expert-educators. (5) Aging studies in Ghana should be attractive to potential service providers.

